# The use of RNA‐based 5'‐aminolevulinate synthase 2 biomarkers in dried blood spots to detect recombinant human erythropoietin microdoses

**DOI:** 10.1002/dta.3123

**Published:** 2021-07-07

**Authors:** Francesco Loria, Holly D. Cox, Sven C. Voss, Angela Rocca, Geoffrey D. Miller, Nathan Townsend, Costas Georgakopoulos, Daniel Eichner, Tiia Kuuranne, Nicolas Leuenberger

**Affiliations:** ^1^ Swiss Laboratory for Doping Analyses, University Center of Legal Medicine, Lausanne and Geneva Lausanne University Hospital and University of Lausanne Lausanne Switzerland; ^2^ Sports Medicine Research and Testing Laboratory Salt Lake City Utah USA; ^3^ Anti‐Doping Lab Qatar Doha Qatar; ^4^ Athlete Health and Performance Centre Aspetar Orthopaedic and Sports Medicine Hospital Doha Doha Qatar

**Keywords:** blood doping, dried blood spots, rhEPO microdoses, RNA‐based biomarkers

## Abstract

The hematological module of the Athlete Biological Passport (ABP) is used for indirect detection of blood manipulations; however, the use of this method to detect doping, such as with microdoses of recombinant human erythropoietin (rhEPO), is problematic. For this reason, the sensitivity of ABP must be enhanced by implementing novel biomarkers. Here, we show that 5'‐aminolevulinate synthase 2 (*ALAS2*) mRNAs are useful transcriptomic biomarkers to improve the indirect detection of rhEPO microdosing. Moreover, the sensitivity was sufficient to distinguish rhEPO administration from exposure to hypoxic conditions. Levels of mRNAs encoding carbonate anhydrase 1 (*CA1*) and solute carrier family 4 member 1 (*SLC4A1*) RNA, as well as the linear (L) and linear + circular (LC) forms of *ALAS2* mRNA, were monitored for 16 days after rhEPO microdosing and during exposure to hypoxic conditions. *ALAS2* mRNAs increased by 300% compared with the baseline values after rhEPO microdosing. Moreover, *ALAS2* mRNAs were not significantly increased under hypoxic conditions. By contrast, *CA1* mRNA was increased after both rhEPO microdosing and hypoxia, whereas *SLC4A1* mRNA did not significantly increase under either condition. Furthermore, the analyses described here were performed using dried blood spots (DBSs), which provide advantages in terms of the sample collection, transport, and storage logistics. This study demonstrates that *ALAS2* mRNA levels are sensitive and specific transcriptomic biomarkers for the detection of rhEPO microdosing using the hematological module of the ABP, and this method is compatible with the use of DBSs for anti‐doping analyses.

## 1 INTRODUCTION

Indirect detection of blood manipulations using the hematological module of the Athlete Biological Passport (ABP) can be challenging, for example, in unmasking doping with microdoses of recombinant human erythropoietin (rhEPO).^1^, ^2^ Developing transcriptomics methods is a promising approach to improve the sensitivity of the hematological module of ABP. A number of studies have attempted to characterize the transcriptomic blood signature after rhEPO administration, and several genes have been identified as potential RNA‐based biomarkers.^3^, ^4^ Among these, mRNAs encoding 5'‐aminolevulinate synthase 2 (*ALAS2*), carbonic anhydrase 1 (*CA1*), and solute carrier family 4 member 1 (*SLC4A1*) have been proposed as candidates for the detection of blood manipulations. In earlier studies, Salamin et al. and Loria et al. demonstrated that the expression of these mRNAs increased following the administration of therapeutic doses of rhEPO.^5^, ^6^, ^7^ Both the linear and the linear + circular forms of *ALAS2* mRNA (*ALAS2 L* and *ALAS2 LC*, respectively) may be useful as potential biomarkers, as both were found to increase after therapeutic injection of rhEPO.^5^ Circular mRNAs have a head and tail joined at the splice site, rendering them more stable than linear mRNAs.^8^ This propriety could be beneficial for monitoring RNA‐based biomarkers in the anti‐doping context.

The large intraindividual variation in the expression of biomarkers is problematic for the detection of blood doping using the ABP. Environmental conditions such as altitude (hypoxia) can introduce an additional challenge. Athletes often live or train at altitude to improve their endurance,^9^ and hypoxia can affect the percentage of reticulocytes (%RET) and the hemoglobin level, which are used by the hematological module, resulting in ABP profiles suspicious for doping.^10^, ^11^, ^12^

Furthermore, the official method recommended by the World Anti‐Doping Agency (WADA) for the collection of whole blood for ABP analysis is venipuncture into ethylenediaminetetraacetic acid (EDTA) tubes. However, this approach involves many obstacles related to sample collection, transport, and storage.^13^ Thus, WADA and different research groups have tested other matrices with the aim of improving the logistics of sample handling. The use of dried blood spots (DBSs), which are already used in pediatric science to collect blood from newborns, could resolve some issues around ABP sample handling.^14^ DBSs do not need to be collected by trained medical personnel and can also be transported and stored at room temperature.^15^ DBSs were already used in anti‐doping studies for detection of blood transfusion^16^ and direct detection of rhEPO.^17^ Moreover, previous studies done by our group on RNA‐based biomarkers for indirect detection of rhEPO use demonstrate that *ALAS2 LC*, *ALAS2 L*, *CA1*, and *SLC4A1* mRNAs can be detected using DBSs.^5^, ^6^

The present study confirms that RNA‐based biomarkers could offer a viable solution for increasing the sensitivity and specificity of the ABP. Furthermore, the method reported here provides a solution for distinguishing between variations due to rhEPO use and variations due to hypoxia exposure using DBSs as the matrix.

## 2 MATERIALS AND METHODS

### 2.1 Clinical study of rhEPO microdosing

Changes in RNA‐based biomarkers after microdoses of rhEPO were analyzed in DBS samples from a previous clinical study (ClinicalTrials.gov, NCT04073849). Briefly, 21 healthy male athletes were blinded and separated into two cohorts, rhEPO‐treated group and control group. In the first phase of the study, the treated participants received eight injections of a boosting dose (40 IU/kg, s.c.) of rhEPO (epoetin alfa, EPOGEN®, USA) over 20 days. In the second part of the study, after a 10‐day washout period, the rhEPO‐treated subjects received eight injections of an rhEPO microdose (900 IU, ~13 IU/kg) over 12 days. Volunteers in the control group received injections of a saline solution instead of rhEPO at the same delivery method and frequency.

### 2.2 Clinical study of hypoxia

In addition, samples from a controlled cross‐over trial performed by Voss et al.^10^ were used to monitor transcriptomic biomarkers under hypoxic conditions. In brief, 10 healthy male athletes were separated into two groups, altitude (ALT) and control (CON). For the ALT protocol, volunteers were exposed to hypoxic conditions in altitude‐simulating rooms (14 days; 10 h per night at a simulated altitude of 3000 m and 6 h per day at a simulated altitude of 5400 m). Subjects in the control group stayed in the same rooms, but the simulated altitude remained at 250 m.

### 2.3 RNA extraction and analysis

mRNA was extracted manually from DBSs and analyzed as described by Loria et al.^6^ using the miRNeasy Mini Kit (Qiagen, Germany), following the manufacturer's instructions with minor modifications.^5^, ^6^ After being cut from the collection card, the whole DBS was transferred into a 2‐ml conical polypropylene microcentrifuge tube (Eppendorf, Switzerland). The cells were lysed by adding 1‐ml QIAzol lysis reagent (Qiagen) to the tube and agitated (450 rpm) at 37°C for 15 min. The tube was then sonicated (Sonicator S30®, Elmasonic®, Germany) for 15 min and agitated again for 15 min under the same conditions as in the first agitation step. Subsequently, chloroform (250 μl) was added, and the sample was vortexed, incubated at room temperature for 5 min, and then centrifuged for 15 min at 12,000 × *g* for 15 min. The aqueous phase (525 μl) was transferred to a new 2‐ml microcentrifuge tube and mixed with 800‐μl ethanol and then transferred onto an RNeasy Mini spin column for washing. The extracted RNA was eluted with 50‐μl RNase‐free water.

Transcriptomic biomarkers were analyzed by reverse transcription‐quantitative polymerase chain reaction (RT‐qPCR). Freshly extracted RNA was first reverse‐transcribed into complementary DNA (cDNA) using the Transcriptor First Strand cDNA Synthesis Kit (Roche Life Science, Switzerland). The cDNA was diluted 1:10 in H_2_O, and 4 μl of sample was loaded manually into 384‐well plates (Roche Life Science, Switzerland). A 6 μl of a primer mix consisting of 240‐μl SYBR Green Master Mix (Qiagen) and 48 μl of primers targeting the reference or target genes was added to wells with cDNA samples. The primers were prepared by MicroSynth based on sequences previously used by our group.^5^, ^6^, ^18^ All samples were analyzed in triplicate. The loaded plate was spin down at 2000 rpm for 2 min prior to RT‐qPCR analysis on the LightCycler 480 System (Roche Life Science) with the following cycles: 1 cycle of denaturation 10 min at 95°C, 45 cycles of amplification 10 s at 95°C and 1 min at 60°C, and 1 cycle of melting curve 1 min at 55°C and 5 s at 95°C and cooling down to 40°C during 30 s. Results obtained for the target mRNAs (*ALAS2 LC*, *ALAS2 L*, *CA1*, *SLC4A1*) were normalized using the mean of the Cq values of three reference mRNAs (*GAPDH, RGCC L*, and *RGCC C*). Results were analyzed using LightCycler software (Version 1.5.0.39).

### 2.4 ALAS2 and CA1 proteins measurement

ALAS2 and CA1 proteins were measured with respective ELISA according to manufacturer's instructions (Human ALAS2 ELISA, Abbexa Ltd., Switzerland) and (Human CA1 ELISA: LSBio, USA).

### 2.5 Statistical analysis

To compare the %RET with values obtained by RT‐qPCR for transcriptomic biomarkers, data were set to the percentage at baseline (%baseline). The normality of the data was determined with the Shapiro test. *T*‐tests were used to compare differences between treated (rhEPO and hypoxia) and nontreated samples. *P* < 0.05 was considered statistically significant. Statistical analyses were performed with R software (R Studio Version 1.3.959).

## 3 RESULTS

**FIGURE 1:**
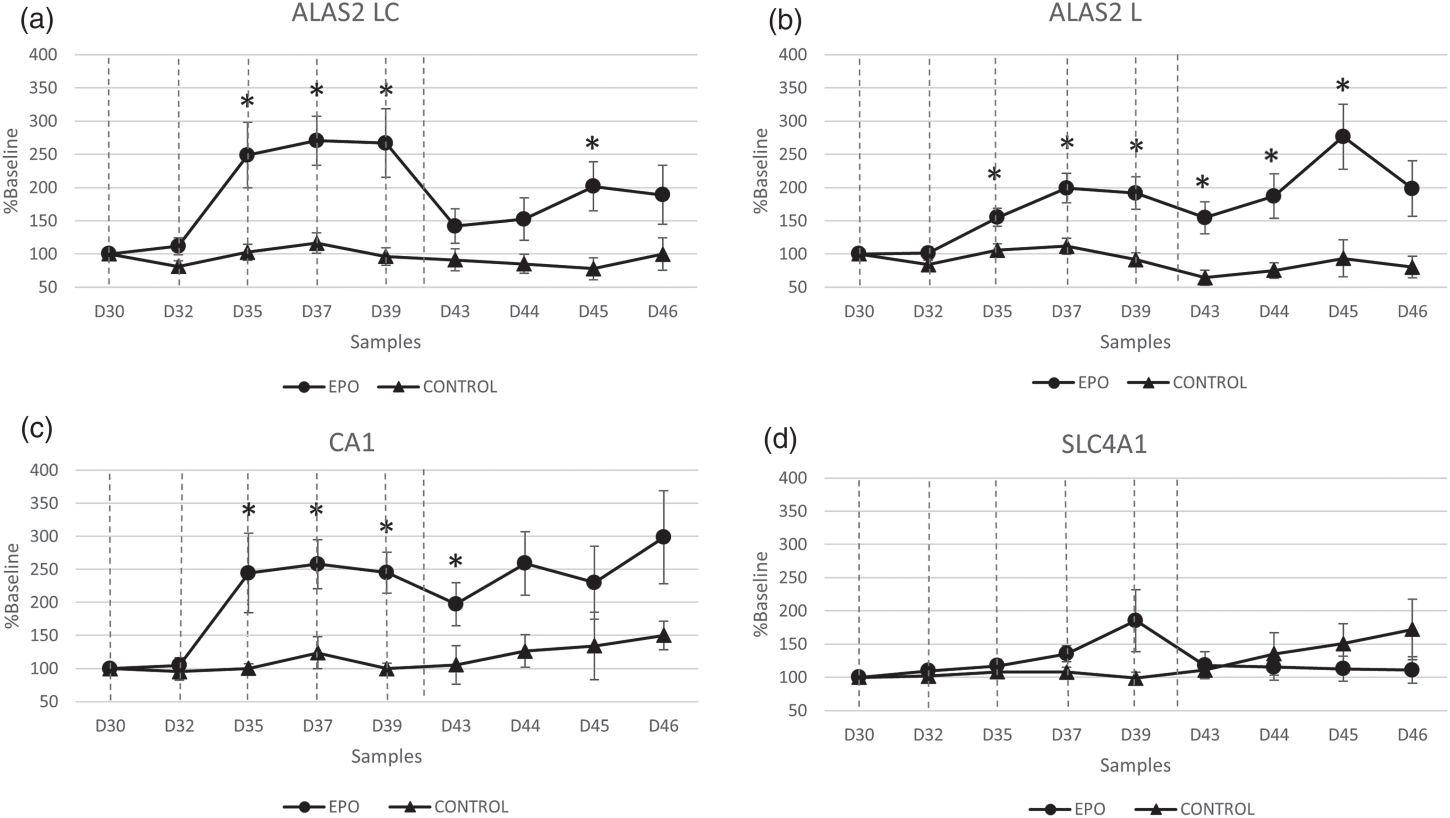
The effects of rhEPO micro‐doses on selected mRNA levels. The levels of *ALAS2 LC* (a), *ALAS2 L* (b), *SLC4A1* (c), and *CA1* (d) mRNAs in DBS samples after rhEPO (treated) or saline (control) microdosing, expressed as a percentage of the baseline values. *P < 0.05 versus control. The dashed lines indicate injections of rhEPO microdoses (900 IU)

When compared with the baseline, the levels of *ALAS2 LC*, *ALAS2 L*, *CA1*, and *SLC4A1* mRNAs increased significantly after the rhEPO boosting doses (Figure S1), which supports previously published findings.^5^, ^6^ In addition, the levels of *ALAS2 LC*, *ALAS2 L*, and *CA1* mRNAs were significantly higher after rhEPO microdosing (Figure 1). After microdosing, the expression of *ALAS2 LC* mRNA increased by up to 300% from the baseline value at Day 35 and was significantly different than the levels in the control group at multiple time points (D35, *P* = 0.01; D37, *P* = 0.002; D39, *P* = 0.009; and D45, *P* = 0.01) (Figure 1a). Similarly, rhEPO microdosing increased the level of *ALAS2 L* mRNA by up to 300% from the baseline value, with significant differences from the control group at D35 (*P* = 0.01), D37 (*P* = 0.004), D39 (*P* = 0.002), D43 (*P* = 0.005), D44 (*P* = 0.01), and D45 (*P* = 0.009) (Figure 1b). The level of *CA1* mRNA was also increased by up to 300% after rhEPO microdosing, with significant differences from the control group at D35 (*P* = 0.04), D37 (*P* = 0.009), D39 (*P* < 0.001), and D44 (*P* = 0.03) (Figure 1c). By contrast, the expression of *SLC4A1* did not differ significantly between the control and rhEPO microdosing groups. In the analysis, the range of Cq values obtain during RT‐qPCR are for *GAPDH* (24–28), *RGCC L* (25–30), *RGCC C* (25–31), *ALAS2 LC* (22–30), *ALAS2 L* (22–30), *CA1* (22–30), and *SLC4A1* (26–31).

**FIGURE 2:**
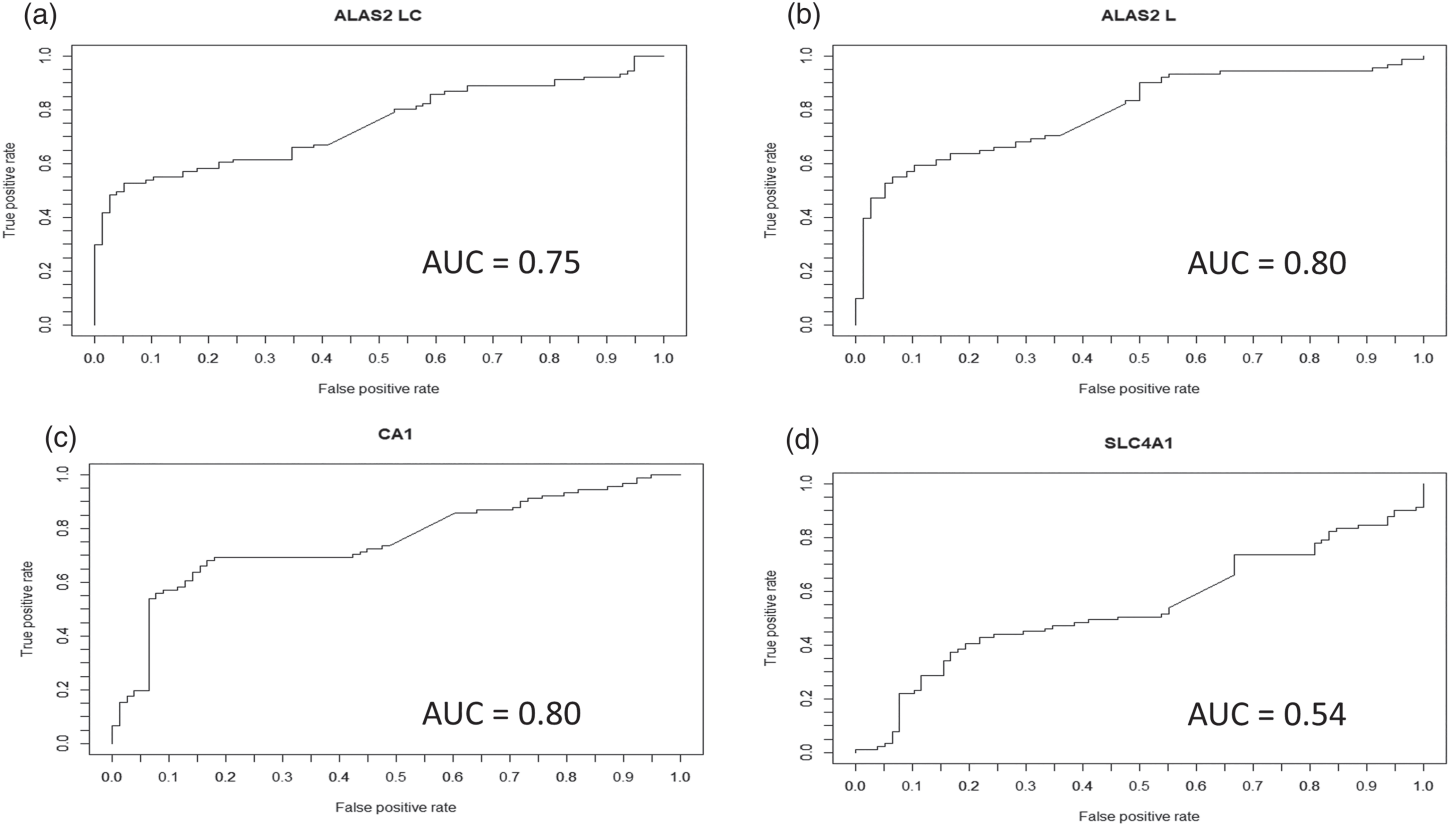
The abilities of the RNA biomarkers to detect rhEPO micro‐dosing. The sensitivities and specificities of *ALAS2 LC* (a), *ALAS2 L* (b), *CA1* (c), and *SLC4A1* (d) mRNAs for the identification of rhEPO microdose injection (900 IU)

Next, the specificities and sensitivities of these potential RNA biomarkers for the detection of rhEPO microdosing were investigated. The *ALAS2 LC* mRNA level had a sensitivity of >50% when the specificity was >95% and had an area under the curve (AUC) value of 0.75 (Figure 2a). For the *ALAS2 L* mRNA level, the sensitivity was 60% at a specificity of >95%, and the AUC value was 0.80 (Figure 2b), whereas the *CA1* mRNA level had a sensitivity of >50% at >95% specificity and an AUC value of 75% (Figure 2c). Finally, the *SLC4A1* mRNA level reached 50% sensitivity when the specificity was <50%, and the AUC value was 0.54 (Figure 2d). Moreover, maximum individual increase after injection of microdoses was 450% for *ALAS2 LC*, 400% for *ALAS2 L*, 350% for *CA1*, and 300% for *SLC4A1* (Figure S2).

Finally, the effect of rhEPO on ALAS2 and CA1 protein expression was monitored in two subjects. Unlike the mRNA levels, the ALAS2 and CA1 protein levels were not affected by the administration of rhEPO boosting doses (Figure S3).

**FIGURE 3:**
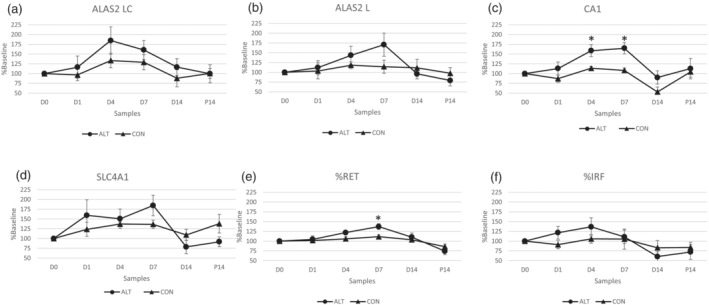
The effects of hypoxia on selected mRNA levels. The levels of *ALAS2 LC* (a), *ALAS2 L* (b), *CA1* (c), and *SLC4A1* (d) mRNAs in DBS samples after hypoxia exposure, expressed as a percentage of the baseline values. *P < 0.05 versus CON group

Analyses of samples from the ALT and CON groups revealed that hypoxia showed only a small and statistically insignificant effect on the expression of *ALAS2 LC*, *ALAS2 L*, and *SLC4A1* mRNAs (Figure 3a,b,d). By contrast, the level of *CA1* mRNA was significantly higher in the ALT group compared with the CON group at D4 (*P* = 0.02) and D7 (*P* = 0.007) (Figure 3c). Moreover, exposure to hypoxia had a significant effect on the %RET, a component of the ABP (D7, *P* = 0.01) (Figure 3e). Notably, this difference did not occur in the immature fraction of RETs (IRF) (Figure 3f).

## 4 DISCUSSION

The identification of rhEPO microdosing using the ABP is challenging because microdoses do not induce significant changes in the %RET or other hematological markers.^2^ Transcriptomic biomarkers are being considered as a potential approach to increase the sensitivity of the ABP. Previous studies have demonstrated that blood manipulations and injection of rhEPO at therapeutic doses induce significant increases in RNA‐based biomarkers, including *ALAS2 LC*, *ALAS2 L*, *CA1*, and *SLC4A1* mRNAs.^5^, ^6^, ^7^ This current study confirmed these findings by demonstrating significant increases in the levels of *ALAS2 LC*, *ALAS2 L*, *CA1*, and *SLC4A1* mRNAs after boosting doses of rhEPO. Moreover, unlike the %RET,^2^ *ALAS2 LC*, *ALAS2 L*, and *CA1* mRNA levels were significantly elevated after rhEPO microdosing. Receiver operating characteristic (ROC) curve analyses, which was carried out to study the characteristics of the response of the selected, demonstrated that these three RNA biomarkers had a specificity of >95% with >50% sensitivity for the identification of rhEPO microdosing. Moreover, AUC was higher than 0.75 and thus comparable to other anti‐doping markers.^19^, ^20^ By contrast, *SLC4A1* mRNA levels were not significantly altered by microdosing, and an ROC curve analysis confirmed poor specificity at a sensitivity of ≥50% (Figure 2). Hypoxic training performed under natural (altitude training) or artificial conditions (hypoxic rooms with decreased oxygen content) is a legal way to stimulate erythropoiesis in order to increase endurance performance. It is frequently used by athletes and has been demonstrated to increase the %RET, which makes hypoxia an important confounding factor to be considered when interpreting hematologic ABP profiles.^10^, ^11^, ^12^ In fact, when only the %RET is used as a biomarker, it is difficult to differentiate between increases caused by hypoxia and those caused by the abuse of rhEPO microdoses. The use of transcriptomic biomarkers could improve the interpretation of hematologic profiles in such a case. As demonstrated in this study, the magnitude of the change in *ALAS2 LC* and *ALAS2 L* and *CA1* mRNA expression after rhEPO microdosing was up to 110% higher than the increase in expression during hypoxic exposure, and the only mRNA that was significantly affected by hypoxia was *CA1*. Moreover, when expression is analyzed individually, it reaches peak to 450% for *ALAS2 LC* after microdose administration (Figure S2). Furthermore, the analyses presented here were performed using DBSs, which were transported from Salt Lake City (USA) and Doha (Qatar) to Lausanne (Switzerland) in an envelope at room temperature. This ease of transport is one of the advantages of DBSs for anti‐doping analyses.^15^ The results presented here indicate that DBSs could be used for transcriptomic^5^, ^6^ or other analyses (such as proteomic analyses) to detect biomarkers of blood manipulations.^16^, ^21^ The data from two volunteers demonstrated that, unlike the expression of the corresponding mRNAs, the levels of ALAS2 and CA1 protein were not affected by rhEPO boosting doses (Figure S3). This may be because ALAS2 protein is present in all red blood cells (RBC) and is found at particularly high concentrations (mg/ml) in erythrocytes^22^; hence, detection of small variations may be difficult, which is not the case of *ALAS2* RNA present only in immature RBC (RET and immature RET), which is a benefit for transcriptomic markers. The level of protein has been measured using two specific ELISA kits.

## 5 LIMITATIONS

Regarding the application of the current results to routine, it has to be noted that until now, all the samples used for this study were collected from male volunteers who participated in the microdose study and for which the results will be presented in the further summary of the still ongoing project. Moreover, hypoxic conditions were simulated in a hypoxic room, and a very high degree of hypoxia was simulated in this study. Further limitation is also the lack samples after hypoxic period, and only one samples has been collected 14 days after hypoxic period. Based on these limitations, the next steps will be to monitor variations in RNA‐based biomarkers in women and to evaluate the effects of more natural hypoxic conditions on transcriptomic markers using real altitude conditions and not hypoxia simulation rooms. Additionally, the combined effects of rhEPO injection and hypoxia on these novel markers will be evaluated, as has been done by Bejder et al.^12^ for markers used in the hematological module of the ABP. Moreover, reproducibility, standardization, and robustness of the method will be tested in collaboration with other anti‐doping laboratories in future studies. Finally, direct detection on microdose samples from Salt Lake City study could be of analytical interest and fitness‐for‐purpose study using the recent published method.^23^, ^24^ As the near‐future perspective, yet importantly, differences in *ALAS2 LC* and *ALAS2* kinetics that could be due to differences in posttranscriptional modifications^25^ will be investigated.

In the future, *ALAS2* mRNA‐based biomarkers could be used to create an alternative OFF‐score in combination with hemoglobin values to provide an additional tool to evaluate suspicious profiles. Moreover, using DBSs for *ALAS2* mRNA analysis allows samples to be re‐analyzed a long time after collection.

## 6 CONCLUSION

In conclusion, this study demonstrates that transcriptomic biomarkers, in particular *ALAS2* mRNAs, could be used to improve the sensitivity and specificity of the hematologic module of the ABP and are compatible with the use of DBSs for anti‐doping analyses. The expression of *SLC4A1* mRNA was increased by boosting/therapeutic doses of rhEPO, but not microdoses, indicating that it is not a useful biomarker to detect microdosing. *CA1* mRNA is an interesting candidate biomarker, although, unlike *ALAS2* mRNA, its expression also increased significantly following exposure to hypoxia.

## Supporting information


**Figure S1:** The effects of rhEPO boosting doses on selected mRNA levels as a percentage of the baseline. Induction of *ALAS2 LC* (A), *ALAS2 L* (B), *CA1* (C), and *SLC4A1* (D) mRNAs, expressed as a percentage of the baseline values. *P < 0.05 versus baseline. The dashed lines indicate injections of rhEPO boosting doses (40 IU/kg).

**Figure S2:** %Baseline results after injections of micro doses on three individual subjects Baseline results of *ALAS2 LC* (A), *ALAS2 L* (B), *CA1* (C), *SLC4A1* (D). In three subjects after rhEPO micro doses, expressed as a percentage of the baseline values. The dashed lines indicate injections of rhEPO micro doses (900 IU).

**Figure S3:** The effects of rhEPO boosting doses on ALAS2 and CA1 mRNA and protein levels. The levels of the *ALAS2 LC* mRNA and ALAS2 protein (A and B) and *CA1* mRNA and CA1 protein (C and D) in two subjects after rhEPO boosting doses, expressed as a percentage of the baseline values. The dashed lines indicate injections of rhEPO boosting doses (40 IU/kg).
