# Supplementary figures and images for: The use of RNA‐based 5'‐aminolevulinate synthase 2 biomarkers in dried blood spots to detect recombinant human erythropoietin microdoses
Source: Drug Test Anal. 2021 Jul 7;14(5):826–32. doi: 10.1002/dta.3123 (PMC9545850; doi:10.1002/dta.3123)

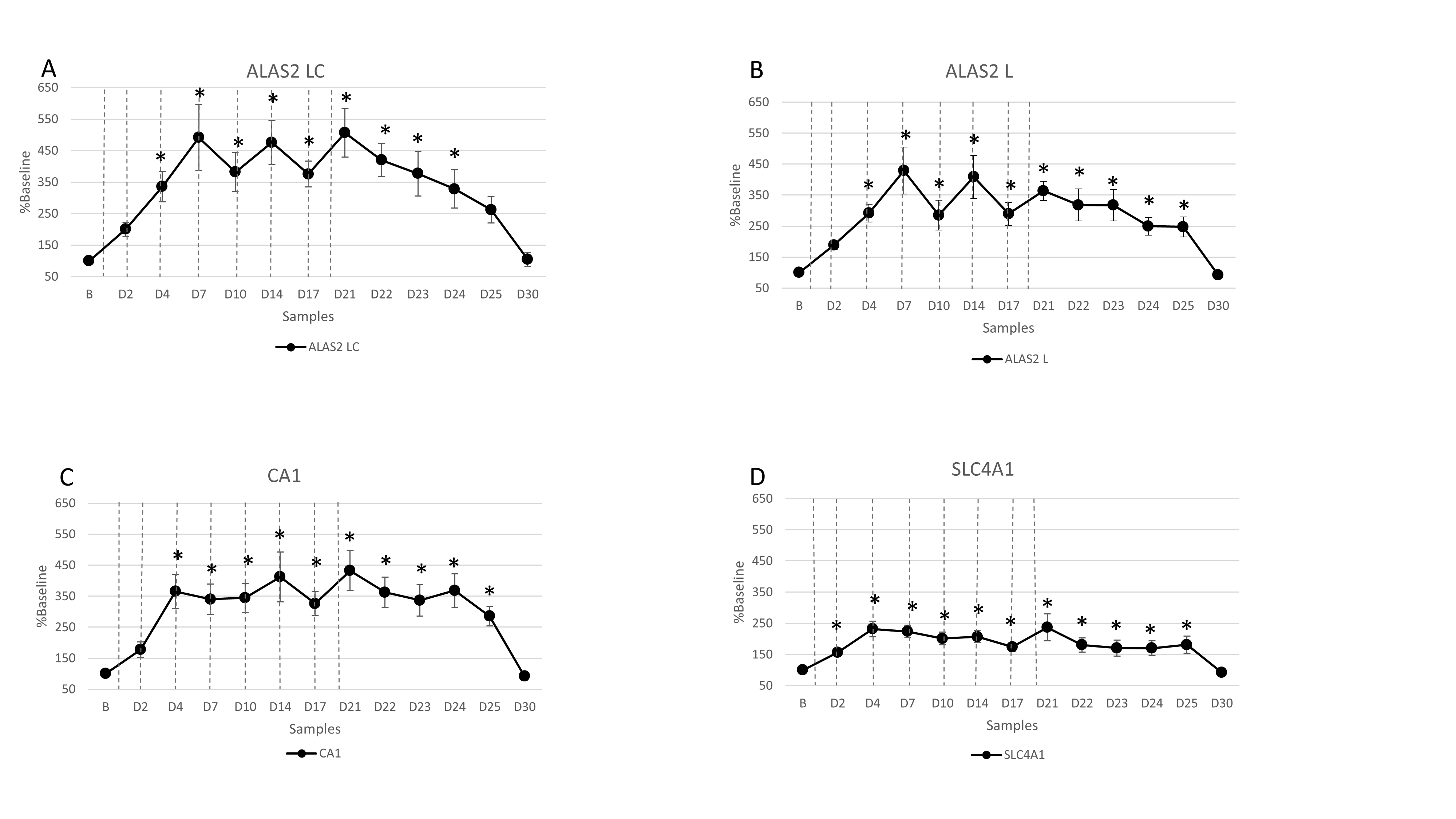

Supplement: Supplementary file 1 — Figure S1: The effects of rhEPO boosting doses on selected mRNA levels as a percentage of the baseline. Induction of ALAS2 LC (A), ALAS2 L (B), CA1 (C), and SLC4A1 (D) mRNAs, expressed as a percentage of the baseline values. *P < 0.05 versus baseline. The dashed lines indicate injections of rhEPO boosting doses (40 IU/kg). [file DTA-14-826-s001.tiff]

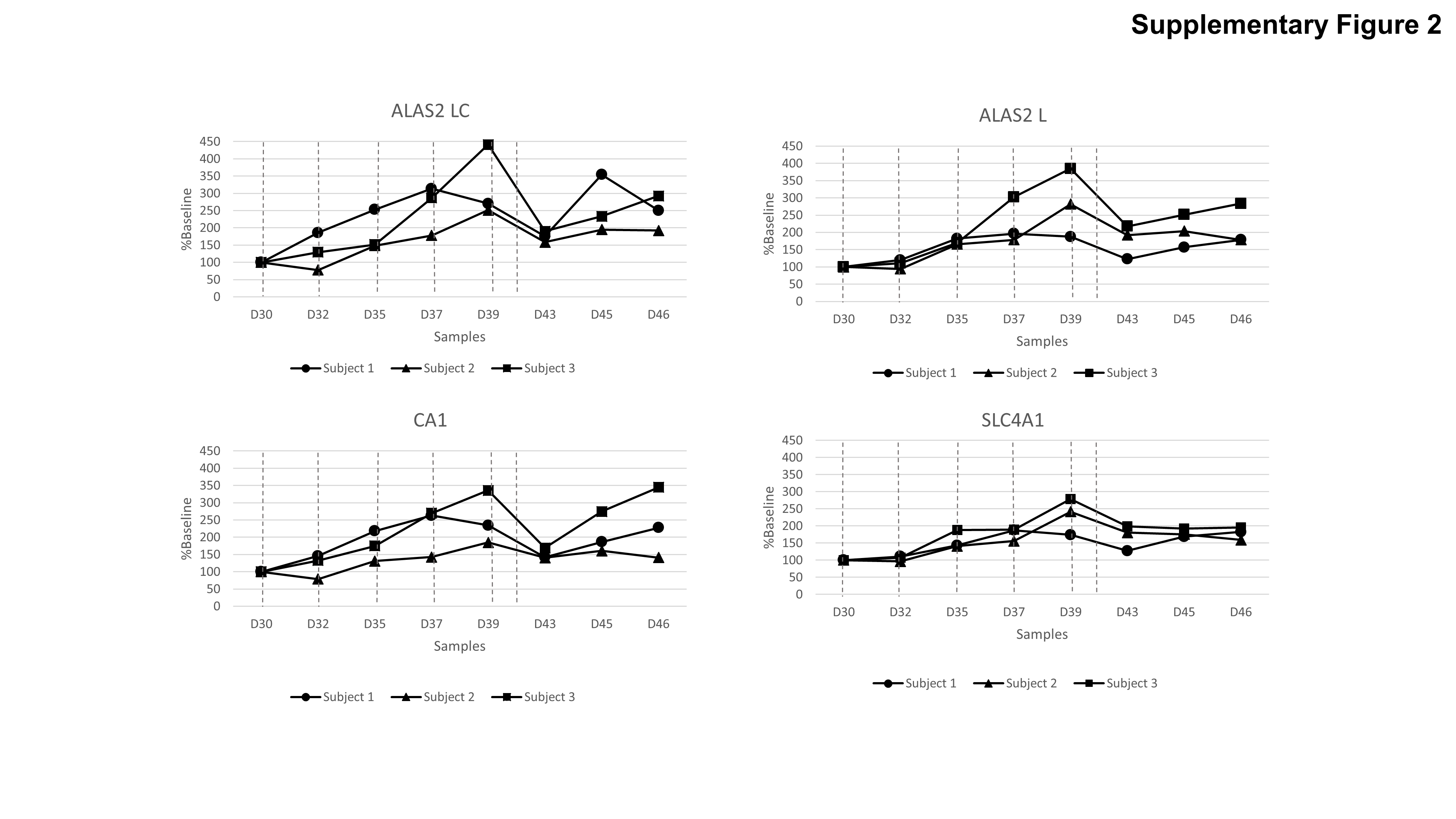

Supplement: Supplementary file 2 — Figure S2: %Baseline results after injections of micro doses on three individual subjects Baseline results of ALAS2 LC (A), ALAS2 L (B), CA1 (C), SLC4A1 (D). In three subjects after rhEPO micro doses, expressed as a percentage of the baseline values. The dashed lines indicate injections of rhEPO micro doses (900 IU). [file DTA-14-826-s003.tiff]

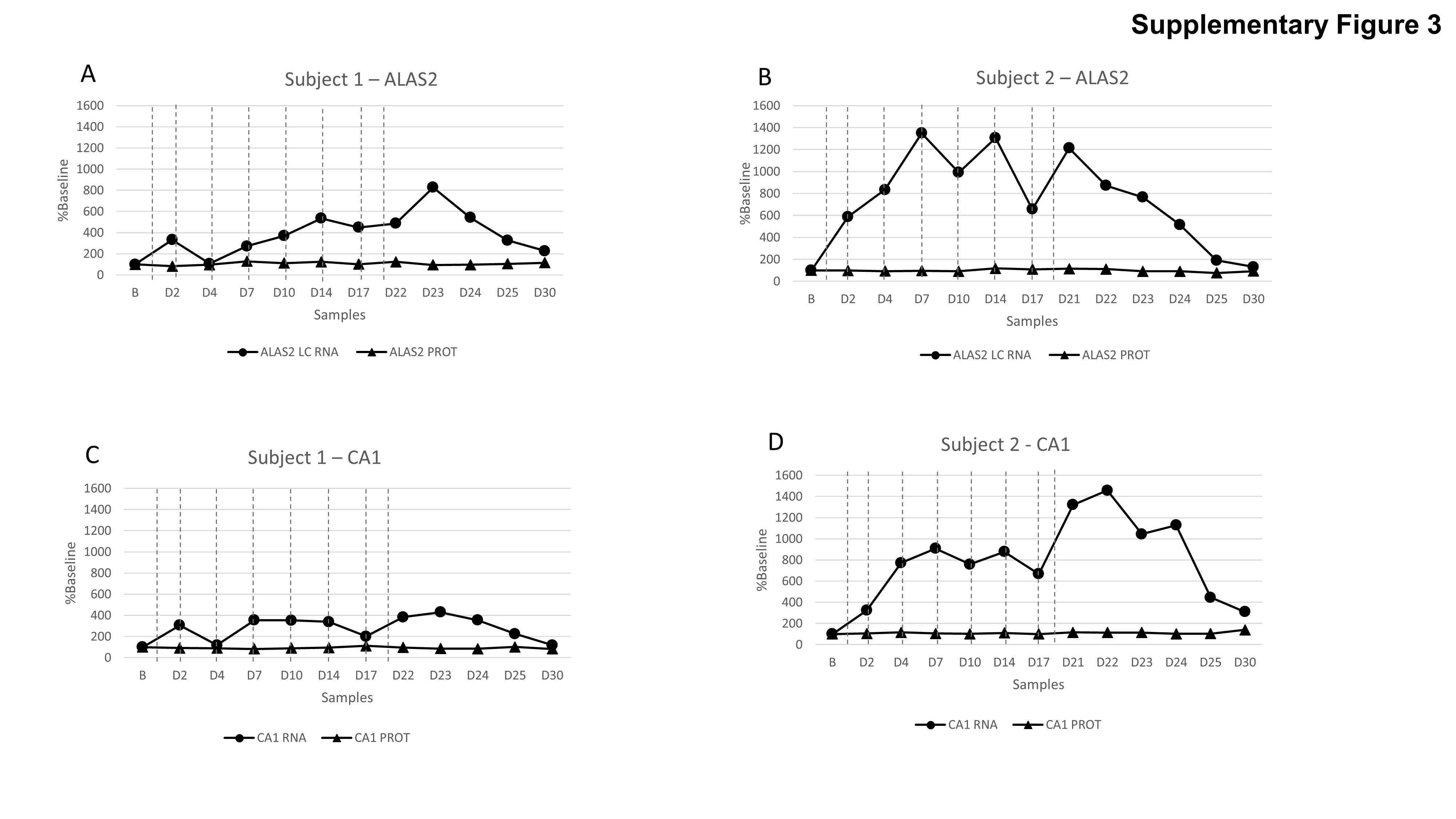

Supplement: Supplementary file 3 — Figure S3: The effects of rhEPO boosting doses on ALAS2 and CA1 mRNA and protein levels. The levels of the ALAS2 LC mRNA and ALAS2 protein (A and B) and CA1 mRNA and CA1 protein (C and D) in two subjects after rhEPO boosting doses, expressed as a percentage of the baseline values. The dashed lines indicate injections of rhEPO boosting doses (40 IU/kg). [file DTA-14-826-s002.tiff]
